# Identification of influencers through the wisdom of crowds

**DOI:** 10.1371/journal.pone.0200109

**Published:** 2018-07-16

**Authors:** Radu Tanase, Claudio J. Tessone, René Algesheimer

**Affiliations:** URPP Social Networks, University of Zurich, Zurich, Switzerland; Universidad Rey Juan Carlos, SPAIN

## Abstract

Identifying individuals who are influential in diffusing information, ideas or products in a population remains a challenging problem. Most extant work can be abstracted by a process in which researchers first decide which features describe an influencer and then identify them as the individuals with the highest values of these features. This makes the identification dependent on the relevance of the selected features and it still remains uncertain if triggering the identified influencers leads to a behavioral change in others. Furthermore, most work was developed for cross-sectional or time-aggregated datasets, where the time-evolution of influence processes cannot be observed. We show that mapping the influencer identification to a wisdom of crowds problem overcomes these limitations. We present a framework in which the individuals in a social group repeatedly evaluate the contribution of other members according to what they perceive as valuable and not according to predefined features. We propose a method to aggregate the behavioral reactions of the members of the social group into a collective judgment that considers the temporal variation of influence processes. Using data from three large news providers, we show that the members of the group surprisingly agree on who are the influential individuals. The aggregation method addresses different sources of heterogeneity encountered in social systems and leads to results that are easily interpretable and comparable within and across systems. The approach we propose is computationally scalable and can be applied to any social systems where behavioral reactions are observable.

## Introduction

Firms, political parties and organisations increasingly rely on engineering social contagion to spread products, ideas or behaviours. Already for more than half of a century researchers have realised that a relatively small number of people can have a great impact on the opinions and behaviour of many others. The concept of opinion leaders (influencers or influentials [1, 2]) was first introduced by Katz [3] in the study of the two step model of communication flow between the mass media and the public and since then it has been revisited in a plethora of studies across many academic disciplines [4–12]. Extensive research has shown that influencers drive new product adoption [4, 6, 8, 9], public health policies [12] or voting behaviour [2]. In consequence, a large body of literature has been devoted to the identification of influencers [7, 13–20], which is still considered today as one of the most important and challenging problems [7, 12, 20].

In general, influencers can be described by a combination of three factors: personification of values (who one is), competence (what one knows) and strategic network location [2, 6]. Most existing identification methods are constructed by selecting one or several features belonging to these factors and identifying the individuals with the highest values of these features. Such features range from psychological traits [2] to expertise [21] or position in the social network (e.g. betweenness centrality [14], eigenvector centrality [13], node accessibility [17], k-shell [16], dynamical influence [18], expected force [19] or collective influence [20]). An important limitation of this kind of approach is that the selection of relevant features is done a-priori by the researcher or practitioner, according to his own subjective preferences and thus the identification of influencers strongly relies on the assumed relevance of the selected features. Furthermore, most proposed features do not incorporate a behavioral reaction of the members of the social group to the actions of the others, which makes it even more difficult to identify a best set of features. Hinz et al. [9] have shown that influencers identified as individuals with either high degree or betweenness centrality are better spreaders of information than individuals with low degree. On the other hand, Watts et al. [5] have shown that except few, rather uncommon cases, influencers identified as central nodes in the social network are not significantly more influential than peripheral ones. This evidence against an universal set of features describing influencers can be explained by the complexity of the influence process. Personal influence has been shown to operate through several latent mechanisms (e.g. contact, socialisation, status competition, social norms) which have a different impact across the five stages of the decision process (knowledge, persuasion, decision, implementation, confirmation) [22, 23]. Under these circumstances, selecting a set of features that describe influencers is difficult without detailed knowledge of the context in which the influence process takes place. Furthermore, most methods can only be applied to a time-aggregated dataset [13, 14, 16–20], which neglects the inherent temporal nature of the influence relationships. There exist several attempts to extend methods to the temporal case [24], but the problem is far from being solved. The widespread belief is that adding a temporal layer leads to much more complex objects, whose study requires the development of sophisticated tools [25, 26].

In this article, we show that mapping the influencer identification problem into a wisdom of crowds one overcomes these limitations. The wisdom of crowds phenomenon [27] was first described by Galton [28] when he observed that social groups can make more accurate collective judgements than expert individuals [27, 28]. Since then, this phenomenon has raised great interest among both researchers and practitioners. People have been shown to make surprisingly accurate judgements when their opinions are aggregated and this concept has been applied to solve a large variety of problems, from prediction markets to informed policy making [27, 29]. The idea also made its way into mainstream applications, being an important mechanism behind creating content on social information sites such as Wikipedia, Quora or Stackoverflow.

We present a framework in which the individuals in a social group repeatedly evaluate the contribution of other members according to what they perceive as valuable and propose a method to aggregate the individual evaluations into a collective judgement. In doing so, we incorporate the behavioral reaction of the social group in the influencer identification. This allows us not to make any assumption on what are the relevant features of the influencers, but to let each individual decide on his own, based on the preferences and beliefs held at that point in time.

The aggregation method we propose: (1) takes the variation of influence process into account; (2) addresses different sources of heterogeneity specific to social systems; (3) leads to results that are interpretable and comparable within and across systems. To illustrate our approach we collected data from online news discussions from three, large independent news providers: CNN, The Atlantic and The Telegraph. We show that following the approach we propose, it is straightforward to reveal those users who are consistently the most influential. The method we propose is computationally scalable and can be applied to any social systems where such behavior reactions can be observed. Our results show that under this mapping, the temporality of the data provides in fact a simplification of the influencer identification. This supports a recent study [25] which shows that temporal complexity may in fact simplify certain problems if seen through the right perspective.

## Materials and methods

### Identification approach

We consider that individuals become influential due to a latent construct they possess which reflects their knowledge and skills, as well as preferences and beliefs. We call this unobserved construct the latent potential to influence. This potential is revealed during social interactions and can be evaluated by other participants through a voting system (e.g. up-votes on discussion platforms). While traditional methods use features set *a priori* by the practitioner or the scientist, our method uses the crowd’s judgement, expressed through votes. In this way we incorporate the behavioral reaction of others into the influencer identification. Operationalising influence in terms of votes reflects both the heterogeneity in skills and knowledge between contributors and the heterogeneity in preferences and beliefs between the evaluators. The latent potential to influence is uncovered by aggregating the individual evaluations. Commonly used methods include the total number of positive evaluations (variations of this are commonly used in social information sites), the mean or the median [28]. However, when applied to systems characterised by a heavy-tailed distribution of variables describing the system (like many social media platforms), such methods might be biased as the quantities they aggregate are not directly comparable. To address all previous shortcomings, we develop the *influence potential (IP)*, a new aggregation method. In the remainder of the article we will use the term *event* to describe a time-window capturing social interactions between individuals. Without loss of generality, the events take place at different points in time, which implies there is a sense of temporality in the data. However, this assumption is not restrictive and the events can also be concurrent. For every event we rank all participants in increasing order of votes received and compute the event rank of an individual in an event as the rank normalised by the total number of participants in the event plus a constant. Formally, the *event rank* of individual *i* in event *t* is defined as *R*_*t*_(*i*) = rank(*i*)/(*n*_*t*_ + *c*), where *n*_*t*_ represents the number of participants in event *t* (*event size* of *t*) and *c* is an additive constant which controls for the event size. Further, let $E^{i}$ be the set of events where *i* participated. The influence potential (IP) of an individual is the mean normalised rank over the events where he participated minus the respective variance. That is, the influence potential of individual *i* is

$$\begin{array}{c} I\left(i\right)={\left\langle R_{t}\left(i\right)\right\rangle }_{t\in E^{i}}-({\left\langle R_{t}{\left(i\right)}^{2}\right\rangle }_{t\in E^{i}}-{\left\langle R_{t}\left(i\right)\right\rangle }_{t\in E^{i}}^{2}). \end{array}$$ (1)

The variance term is introduced to penalise the lack of consistency in the ranks obtained. The IP reflects the extent to which most participants in an event consistently appreciated the contribution of the individual each time he was active. Notice that we do not impose a criteria on how the contribution should be evaluated. The IP is bounded in the interval [0, 1] (see S3 Appendix). A value close to zero is obtained for individuals who either consistently rank low in the votes distribution or have a high variation in the votes score across all the events they participated in. Such individuals have a low potential to influence as, either their contribution is rarely appreciated or this happens with a high level of uncertainty, questioning their inherent abilities. On the other hand, a value close to one can only be obtained for individuals who always collect the most votes in the events they participate. Such individuals have a high potential to influence as, due to some construct we do not directly observe, they always attract the highest evaluation. An implicit assumption made in Eq 1 is that the activity of an individual (defined as the the number of events attended) is not alone informative for the latent potential to influence but is rather an opportunity for the latent potential to influence to be manifested.

### Small sample bias

The IP is at its root a statistical aggregation and, as any statistical measure, is susceptible to bias arising from small samples. This bias can be induced in two ways: (1) if within an event there are few participants; (2) if an individual takes part in a low number of events. In events with few participants (thus implicitly few judges), the IP scores might be biased as they violate one of the critical assumptions behind the wisdom of crowds: a large number of evaluations. To address this we penalise event ranks obtained in small events by introducing the constant term *c* in the event rank normalisation. By changing *c* one can emphasise or diminish the role of the event size in computing the IP. S10 Fig illustrates the impact *c* has on computing the event ranks. For large *c*, high IP values can only be obtained in the limit of large events, while for small *c* the effect of the event size on the event rank is negligible. This has practical implications for studying dynamical processes like information propagation where the size of the susceptible population plays an important role. The second source of small sample bias is the small number of events attended by an individual. In this case the IP might not be informative for the latent potential to influence as by aggregating few data points the results are subjected to randomness. We can address this by setting a threshold on the minimum number of events attended by each individual and remove from the analysis those who attended less. The threshold can be seen as a measure of confidence in the results. The higher the threshold, the higher is the minimum number of events attended by each individual and thus the lower the likelihood that high vote scores are obtained in most events by chance.

## Results

### Data collection

We collected the complete history of online discussions over a long period of time from three large news providers: CNN, The Atlantic and The Telegraph. Such platforms offer an interactive environment in which users have the possibility to express their views, engage in discussions and possibly shape other’s view on the topic. Registered users can post comments in discussion threads and, at the same time, react and evaluate the quality of the posts through a voting tool provided by the platform. The default ordering of the posts on the platform is determined by the number of votes received. The discussions cover a broad range of topics, each thread belonging to one topic category which defines the overall topic of the discussion (e.g. politics, business, etc.). The categories are defined by the news providers and are directly available on the website. Discussion threads for which it was not possible to identify the category have been omitted from the analysis. All platforms are comparable in terms of user experience as they are based on the same technology, provided by Disqus. An overview of the three datasets can be found in Table 1 (approximative figures) and a detailed description of the categories in S1–S3 Tables. In our terminology, the discussion threads represent the events, the contribution of an individual in an event is defined by the total number of posts made in the thread, and the evaluation of the contribution is defined by the number of up-votes received by all posts made in the thread.

**Table 1:** Overview of data analyzed.

Dataset	Period	Active users	Threads	Posts	Cat.
CNN	2012–2014	9 × 10^6^	3 × 10^4^	23 × 10^6^	13
Atlantic	2007–2016	3 × 10^6^	5 × 10^4^	5 × 10^6^	17
Telegraph	2006–2016	5 × 10^6^	33 × 10^4^	22 × 10^6^	15

### Identification of influencers

**Fig 1:**
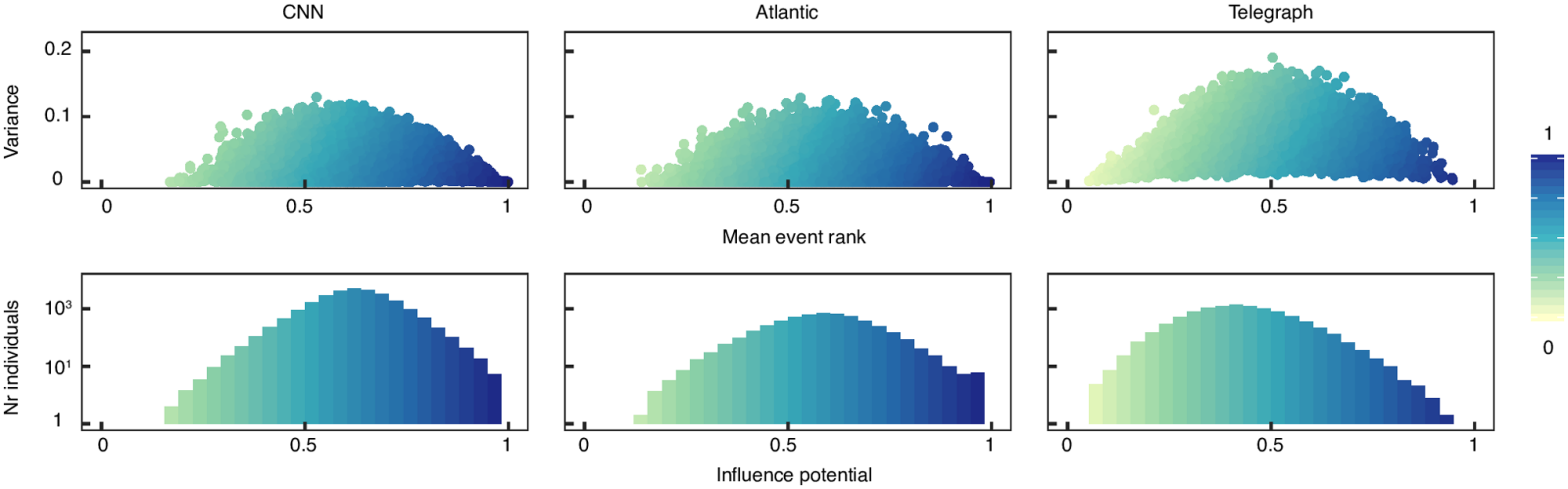
Identification of influencers. Data is pooled from all categories. An individual can be described by multiple data points, each being related to his performance in one category. Number of observations: CNN (115,186), Atlantic (20,136), Telegraph (102,795). The colour of the points is given by the IP. *Upper Panels*: Relationship between the mean event rank (x axis) and the corresponding variance (y axis). There is an inverted U-shape relationship between the mean and the variance of the event ranks. The individuals with high mean and a low variance have the highest IP. *Lower Panels*: Distribution of the IP.

We investigate if for each topic there are individuals who consistently receive most votes each time they are active. In the remainder of the article we use *c* = 1 and consider only individuals who participate in at least 10 events. In S1 Fig we show the results are robust to the choice of *c* and later in the article we show the IP is robust to the number of events observed per individual. In Fig 1, upper panels, we show the relationship between the mean event rank (x axis) and the corresponding variance (y axis). It can be seen there are several individuals with a high mean event rank and a low variance (illustrated with dark blue colour code). In consequence, in the lower panels of Fig 1 we observe a heavy tailed distribution of the IP, with several individuals having high values. This is consistent with existing literature, which states that there are just a few influencers compared to the entire population [5]. This result is rather surprising as we would expect a high disagreement between the participants in an event because what is a valuable contribution is decided by each individual on his own, based on his own preferences and beliefs. We later consider two parsimonious mechanisms and show that none can completely explain the results.

### Zooming in topic categories

**Fig 2:**
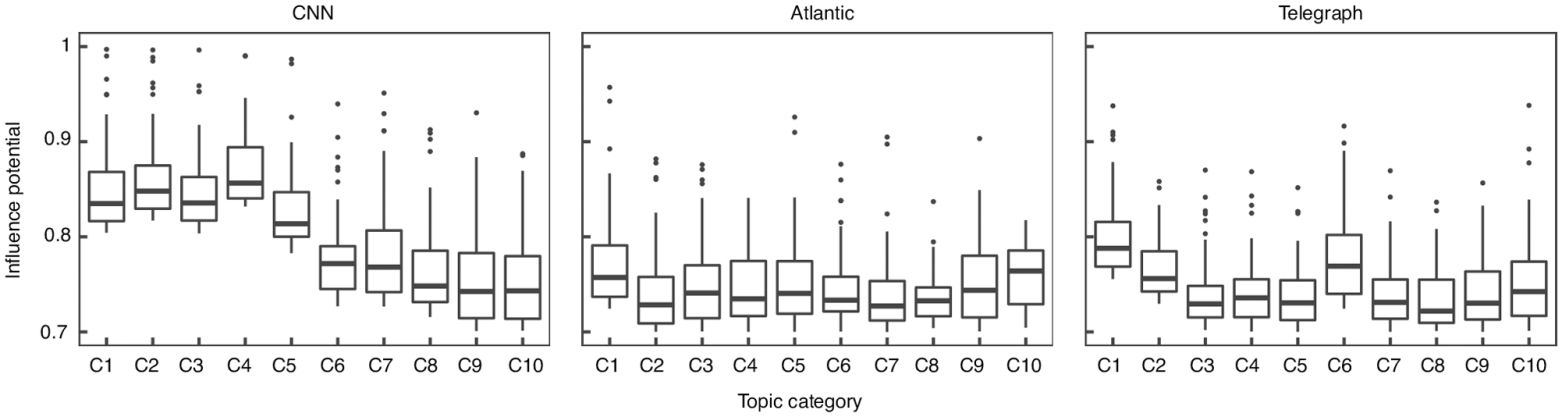
Influencers within topic categories. The x axis represents the topic category. The y axis represents the IP scores of the top 100 individuals with the highest IP within the category. The categories are ordered by the number of users.

**Fig 3:**
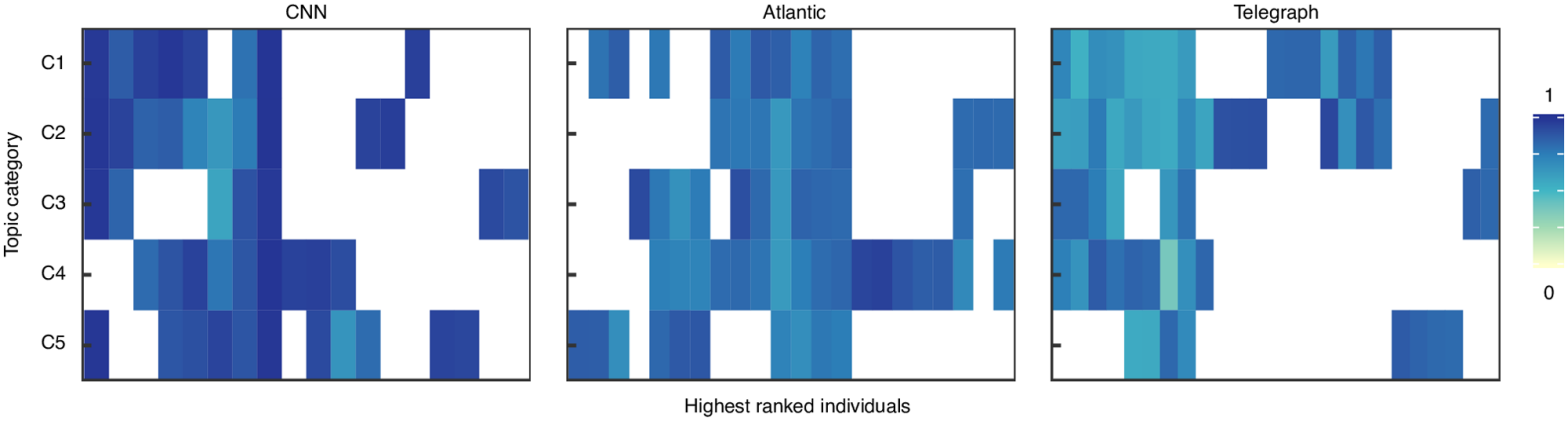
Influencers across categories. The *x* axis represents the IP scores of the top five individuals with the highest IP from the five largest topic categories on each platform. Several individuals appear within top five in more than one category thus the number of observations for each dataset is different (CNN: 18, Atlantic: 22, Telegraph: 25). The *y* axis represents the topic category. If an individual did not participate in a category, it is represented by a blank cell. Most individuals participate in few categories. Individuals who participate in more categories have high IP scores in only few.

We now investigate how the nomination of influencers varies across the topic categories. By doing so, we are able to identify category influencers. Fig 2 contains a boxplot of the highest 100 IP scores within each category. To ease the representation, for each dataset we selected the top ten categories with the highest number of users. We re-labelled each category according to its ranking in terms of number of users among the categories within the same dataset, C1 representing the highest. The list of abbreviations together with the number of users in each category can be found in S1–S3 Tables. Fig 2 shows there is a considerable difference between the highest influencer scores across the different categories (p < 10^−16^, n = 1000, ANOVA test). For example, on the CNN platform, in the first five categories (C1-C5: world, us, opinion, politics, justice) the influencer scores of the top 100 individuals are considerably higher than in the following five (C6-C10: showbiz, tech, health, travel, living). This has practical implications for designing intervention campaigns based on targeting influencers as it shows that in the same system, the extent to which individuals agree on who and what is influential might change depending on the context. Very often in literature it is considered that the extent to which an individual is influential is determined by one or several feature he possesses [2, 6] and it is neglected how, for the same individual, the impact of these features on his perceived influence can vary across different settings. In Fig 3 we selected the top five individuals with the highest IP in the five largest categories and plotted their IP scores across the categories. If an individual did not participate in a category, it was represented by a blank cell in the figure. It can be seen that: (1) most individuals participate in very few categories and (2) individuals who participate in more categories have high IP scores only in few. To explore this effect further, we considered the highest ranked 100 individuals in each of the five categories (*N* = 500) and computed the pairwise Pearson correlation between the scores. S15 Fig shows a high variation in the pairwise correlation between the categories, in all three datasets. Taken together, the results suggest that individuals who are influential across topics are hard to find, possibly because an important component of the latent potential to influence is the *topic expertise* [2]. This finding is in line with early studies which showed that opinion leadership is topic dependent, with different degrees of overlap between the topics [30]. However, in recent studies this is very often neglected as influencers are mostly identified based only on one (often structural) feature [13, 14, 16–20]. Targeting for example a well connected individual who is expert in politics to promote a healthy behaviour has a high risk to fail.

### Different aggregation methods

**Fig 4:**
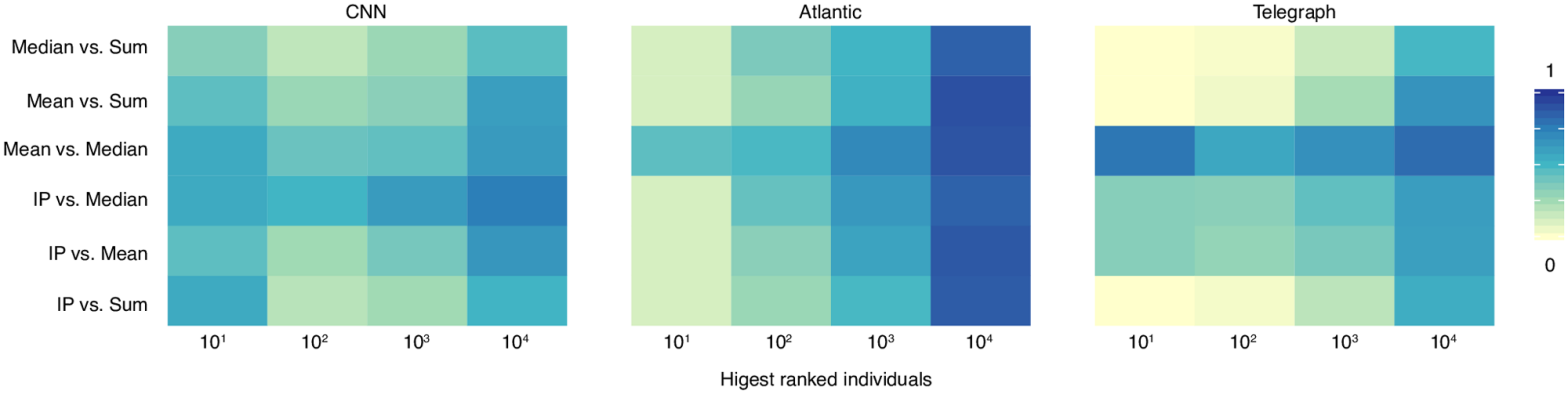
Comparison of results under different aggregation methods. We compare the overlap between the highest ranked individuals by different methods. The x axis represents the number of highest ranked individuals. The y axis represents the overlap between the highest ranked individuals by two methods. Data is pooled from all topic categories. The mean and the median are the most similar. The IP is closest to the median.

We compare our aggregation method against three alternatives often encountered in research or practice: the total number of votes (used regularly on social information sites to rank users), the mean and median [28] number of votes. For every topic, we rank all users according to the four methods and calculate the degree of overlap between the highest ranked users. Fig 4 shows the results. The total number of votes leads to considerably different results, with the lowest overlap with the other methods. One reason is that this method does not control for the difference in activity between individuals nor for the difference in size between events. On the other hand, the highest similarity can be observed between the mean the median. Both methods control for the difference in activity between individuals, but not for the event size. In addition, the median is not sensitive to extreme evaluations which can explain the higher difference observed in the CNN dataset. The IP is closest to the median, with a significant but not high overlap between the two. Compared to existing aggregation methods, the IP has several appealing features.

First, penalizing the mean event rank through the variance term in Eq 1 allows to identify individuals who *consistently* rank high and thus who are consistently outperforming others in obtaining the crowd’s votes.

Second, it addresses different sources of heterogeneity often encountered in social systems. A predominant characteristic of most social systems (including news platforms) is that there is a heavy tailed distribution of the variables describing the system. S2–S4 Figs show there is a large difference in the number of participants in the events. As the total number of votes in an event is proportional to the event size (S5 Fig), it implies that we cannot directly compare the number of votes received in events of different sizes. If we use the mean or the median to aggregate the votes, we are making the implicit assumption that the votes obtained are comparable and thus one vote in an event needs to worth the same as one vote in any other event. However, this assumption is questionable as the events are heterogeneous in the total number of votes cast, even for the same event size (see S5 Fig). Thus, in some events it could be easier to obtain votes than in others. In consequence, an aggregation method like the mean or the median, could be biased towards participation in large events. This is illustrated in S14 Fig, where we can see that for two individuals with the same mean number of upvotes, their ranking in terms of votes obtained within a thread is very different. By using normalized rankings, the IP always considers the vote scores relative to the event, thus controls for the total number of votes cast in the event. In doing so, one vote in an event where few people receive votes worths more that one vote in an event where many people receive votes. Furthermore, the number of events attended by an individual is as well described by a heavy-tailed distribution (S6–S8 Figs). This implies that comparing users in terms of the total number of votes received, as it is done on most social information sites, will favor individuals who are very active. To infer the latent potential to influence, our approach does not take into account the number of events attended (once a minimum number has been achieved). In this way we are, to some extent, separating the tendency of individuals to be active from their latent potential to influence, making the influencer scores comparable across individuals with different levels of activity.

Third, the aggregation method we propose provides normalised results, that are easy to interpret and to compare within and across systems. Individuals who are influential have IP scores close to one, while non-influential individuals have scores close to zero. Because of this, the extent to which somebody is influential can be directly inferred from his IP score, without the need of additional information about the system, like with the other aggregation methods. For example, in order to understand if a certain mean value is high or low, one needs information about the distribution of votes in the events.

### Robustness checks

**Fig 5:**
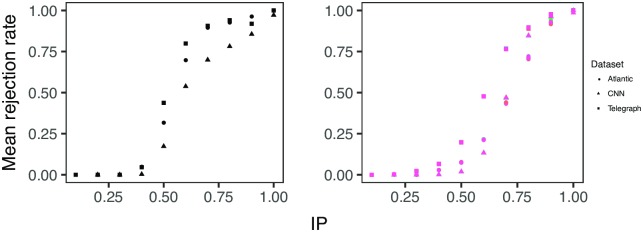
Comparison with null models. The *x* axis represents the IP decile. The *y* axis represents the mean rejection rate at a cutoff value of 5%. The shape is given by the dataset, the color by the preferential attachment parameter *α*. *Left Panel*: Talkativeness null model. *Right Panel*: Preferential attachment null model. For both null models, there is a high mean rejection rate for the highest IP deciles.

Already more that half a century ago, Bass [31] has observed a high correlation between the time a person spends talking and her perceived leadership in the social group. When data is generated by such a mechanism, high IP scores merely reflect *talkativeness* (here defined as the tendency of individuals to post excessively), which is then considered as the main component of the latent potential to influence. To test if data can be explained by the talkativeness effect, we create a null model in which the observed number of votes is uniformly distributed across all posts in a thread (see S1 Appendix). Specifically, for every thread we sample with replacement from all posts a number of times equal to the observed number of votes in the thread. Then we compute the IP as described above, using the sum of randomised votes as input. The procedure is repeated 100 times and the IP under the null model is computed as the mean IP over the repetitions. Under this model, the event rank of an individual in a thread is proportional to his number of posts. Individuals who write more have a higher chance to obtain a high event rank, and thus a high IP. S9 Fig shows that this mechanism can lead to the emergence of individuals with a high IP, even though the allocation of votes is done at random. However, there is little overlap between the highest ranked individuals identified under the two conditions. For the highest ranked 1000 individuals by either IP or the IP under the null model, there is a low Pearson correlation between the scores (cnn: -0.09, atlantic: 0.15, telegraph: 0.23). To explore this effect formally, we conducted a one sample t-test to compare, for each individual, the mean of the IP values simulated from the null model to the observed IP. We tested the null hypothesis that the simulated and the observed IP are equal against the alternative that the simulated IP is less than the observed. To illustrate the results we grouped individuals by deciles of the observed IP (see S4 Table) and computed the mean rejection rate for each decile, at a cutoff value of 5%. The left panel of Fig 5 shows that for low IP values, the rejection rate is close to zero, thus the observed IP values are not different from those generated under the null model. However, for high IP values, corresponding to the identified influencers, the rejection rate is close to one, thus the observed IP values could not have occurred by chance. This implies that in nominating influencers, the crowd considers more (or different) features than just the active participation in the event.

Many social systems are characterised by a rich-get-richer effect [32–34], where individuals who enter the system early have an advantage over those who enter late. This effect is particularly important on many online discussion forums, including our data source, where the default ordering of the posts on the platform is determined by the number of votes received. Such mechanism of sequential voting might lead to herd behavior (e.g. [35]), where a minority of individuals could obtain most votes in a thread, irrespective of their contribution. This is a case when social influence can undermine the wisdom of crowds [36, 37]. Under such a mechanism, high IP scores reflect the ability of an individual to enter the system early and gain initial votes. To test if data can be explained by the rich-get-richer effect, we create a null model in which a vote is allocated with probability *α* to a post selected at random and with probability 1 − *α* to a post selected according to a preferential attachment model in which posts with more votes are more likely to be selected (see S2 Appendix). For *α* ∈ {0, 0.1, …, 1}, for all three datasets the Spearman correlation coefficient between the scores of the highest ranked 1000 individuals by either IP or the IP under the preferential attachment null model is low (*r* <= 0.22). Similarly as above, to explore this effect formally, we conducted a one sample t-test in which we tested the null hypothesis that the simulated and the observed IP are equal, against the alternative that the simulated IP is less than the observed. The right panel of Fig 5 shows that for low IP values, the rejection rate is close to zero, thus the observed IP values are not different from those generated by the null model. However, for high IP values, the rejection rate is close to one, for all values of *α*. This implies that also preferential attachment is not enough to explain high IP results and that the individuals identified by the crowd have unobserved features that allow them to obtain most votes each time they are active. This result is supported by recent work [38], which shows that sequential voting mechanisms can in fact be more efficient for discovering the optimal solutions and alleviates the concern that high IP values are the artifact of a potential preferential attachment mechanism induced by the platform design.

**Fig 6:**
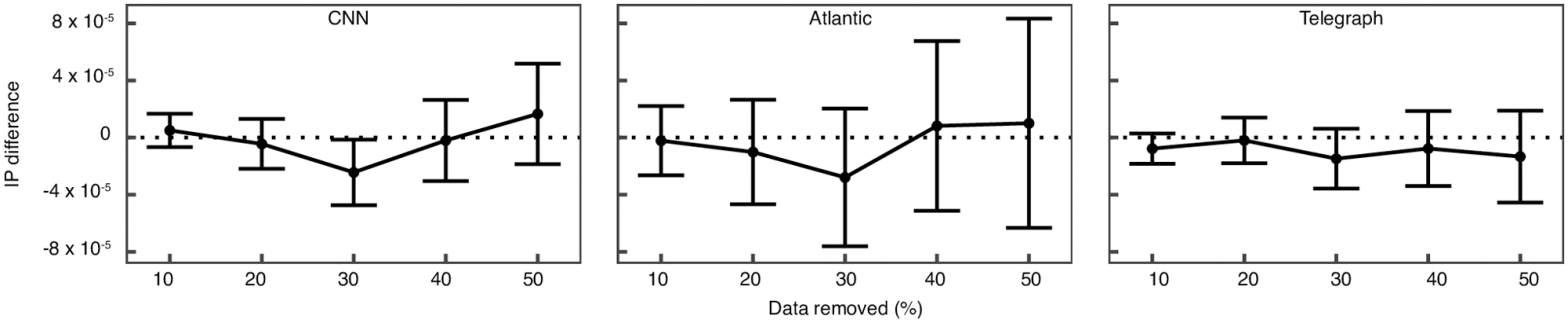
IP scaling with sample size. The *x* axis represents the percentage of events removed at random. The *y* axis represents the mean difference between the IP scores based on the entire sample and the IP scores based on the random subset. Number of observations: CNN (53,393), Atlantic (10,300), Telegraph (59,852). The error bars represent the 95% confidence interval for the mean. Decreasing the sample size does not have a significant effect on the IP.

One concern that might be raised is that high IP values are favored by participation in a low number of events, even after imposing a threshold on the minimum number of events attended. S11 and S12 Figs shows the relationship between the IP and the number of events where an individual participated. For all three datasets, there is a low Pearson correlation between the IP and the logarithm of the number of events (CNN: 0.09, Atlantic: 0.15, Telegraph: 0.09) and a visual investigation of the plots shows there are individuals with high IP values for a wide range of the number of events. To formally show that our results are robust to the sample size we use the following procedure. We define a sequence of percentiles *q* ∈ [0, *q*_*max*_] and for each individual *i* define a set of events $E_{-q}^{i}$ that is constructed by removing at random *q*% of all the events where *i* participated within the topic category. Then compute the IP of *i* over only the events in $E_{-q}^{i}$. For each *q* we repeat the procedure 100 times and compute the difference between the IP based on the entire sample and the mean IP over the repetitions. To ensure the IP is always computed using at least 10 events we remove from the analysis all individuals who did not participate in more than 20 events within a topic category. Fig 6 shows that on average, reducing the sample size at random by even 50% (*q*_*max*_ = 0.5) does not produce higher IP values. In a different analysis, for each individual we sample a fixed number of 10 events (instead of a percentage) from the events where he participated and, similarly as above, compute the IP over the events sampled and repeat the procedure 100 times. For each individual we conduct a one-sample t-test in which we test the null hypothesis that the IP based on the random sample and the observed IP are equal, against the alternative that the IP based on the random sample is greater than the observed. S13 Fig shows the mean rejection rate by IP decile for a cutoff value of 5%. For each decile, in the CNN and The Atlantic datasets the mean rejection rate is close to 5%, while for The Telegraph is slightly higher, around 12%. Taken together, the results show that high IP values are not an artifact of small sample sizes.

## Discussion

Political parties, companies or health organisations are interested to identify influencers and use them as superspreaders of products, ideas or behaviors in intervention campaigns [11, 12, 39]. In the ideal case, to identify influencers researchers would directly observe the actions of the individuals and the causal effect of these actions on the behavior of their peers. However, in most practical applications, both the actions and their effect are not directly observable and thus need to be inferred from an observable quantity. The dominant mindset is to first identify a set of observable features that could best describe an influencer and then look for individuals with high values of these features. While this is a perfectly feasible approach, with a high success across a wide range of applications [6, 8, 12], it also suffers from several drawbacks. First, most proposed features (e.g. [13, 14, 16–20]) are agnostic to the content and context of the social interactions. For example, since a long time centrality in the communication network (defined in various ways, e.g. [13, 14]) has been used as an operationalization of the extent to which someone is an influencer. People who are more central are thought to be more influential. However, this identification is made without observing the content of what is being communicated, the context in which it is communicated and the impact of the content on behavioral actions in the specific context. We believe the importance of these features is both time and context dependent. Who we consider as a reliable source of information might change depending on when we intend to make the decision or its perceived level of risk [12, 40]. Thus this approach is limited by itself, as by construction it can only identify individuals with high values of the selected features, irrespective if these are relevant in the given scenario or not. Second, nowadays datasets are much richer than before, with high time-resolution and detailed individual information being frequently the norm. Classical methods were developed to deal with cross-sectional data, as often researchers and practitioners had a single data snapshot available. There are many attempts to extend these methods to account for increasing levels of complexity like temporal variation, but most often this is not straightforward, leading to complicated mathematical descriptions that are computationally expensive or which come at the price of stronger assumptions, making it difficult to apply them in real-time environments.

In our study we take a different approach and operationalize influence as the behavioral response of an individual to the actions of another. This operationalization gives us the flexibility of not making a priori assumptions on what is the right quantity that measures influence across all different contexts in a system, but allows us to measure the response of each individual, according to his own preferences and beliefs at a given point in time. In doing so, we follow a recently proposed path [25, 26] and show that more complex information can actually simplify the analysis if seen trough the appropriate lens. Influencer identification in temporal systems with a measurable outcome of social interactions (e.g. social media platforms) can be mapped to a wisdom of crowds problem, where individuals decide on their own what is relevant for them at every point in time. By aggregating the individual evaluations, it is straightforward to reveal who is consistently the most influential each time he is active.

The results of data analysis show that indeed influencer identification is highly context-dependent. Individuals who are influential in one topic are seldom influential in others (see Fig 3). This raises a caveat often encountered in social influence studies: the IP can only identify people who are likely to show influence in the context in which the analyzed social interactions took place. Thus, by analyzing online political discussions, we can only identify individuals who are likely to be influential in future online political discussions and any extrapolation of this result outside the context in which the measurement was made has a high degree of uncertainty. In our attempt to keep the aggregation method simple and intuitive, we did not consider that the evaluation received by an individual might be influenced by the individuals against whom he is competing. In discussion threads where many influencers participate, it might be more difficult to obtain a high evaluation due to competition dynamics. An extension of the method to account for such cases might provide a valuable contribution. Furthermore, comparing the influencer scores across different topic categories (see Fig 2) shows there is a difference in the extend to which the crowds agrees on who are the influential individuals. Understating which are the factors that drive these differences might provide an important avenue for future research. We conclude by mentioning that the applicability of the aggregation method is not restricted to the wisdom of crowds scenario. In particular, it could be applied to quantify performance in any temporal system where a performance metric is measured over time. It could be used in diverse disciplines like network science to quantify centrality in temporal networks, management to quantify performance of employees or sports to identify the most valuable players.

## Supporting information

S1 Table CNN dataset. Overview of the topic categories.
(CSV)

S2 Table The Atlantic dataset. Overview of the topic categories.
(CSV)

S3 Table The Telegraph dataset. Overview of the topic categories.
(CSV)

S4 Table Number of users by IP decile. (CSV)

S1 Fig The influence potential is robust to the choice of c. The diagonal panels show the choice of *c* used to computed the IP. The lower triangle panels show the pairwise scatterplots between the IP scores computed with different *c*. The upper triangle panels show the corresponding Pearson correlation coefficient. Data is pooled from all topic categories. An individual can be described by multiple data points, each representing his IP in a category where he participated in at least 10 events. Number of observations: CNN (115,186), Atlantic (20,136), Telegraph (102,795). For all three datasets there is a very high pairwise correlation (Pearson *r* ≥ 0.82) between the IP values for different choices of c.
(PDF)

S2 Fig Distribution of the event size within the topic categories: CNN dataset. (PDF)

S3 Fig Distribution of the event size within the topic categories: Atlantic dataset. (PDF)

S4 Fig Distribution of the event size within the topic categories: Telegraph datataset. (PDF)

S5 Fig Relationship between the event size and the total number of votes in the event. The x axis represent the total number of votes in the event. The y axis represents the event size. Data is pooled from all categories. Number of observations: CNN (31,035), Atlantic (46,639), Telegraph (268,895). The Spearman correlation coefficient *r* is computed for the log values. There is a high correlation between the event size and the total number of votes in the event.
(PDF)

S6 Fig Distribution of the number of events in which individuals participated: CNN dataset. (PDF)

S7 Fig Distribution of the number of events in which individuals participated: Atlantic dataset. (PDF)

S8 Fig Distribution of the number of events in which individuals participated: Telegraph dataset. (PDF)

S9 Fig Distribution of the IP under the talkativeness null model. Data is pooled from all categories. An individual can appear in more than one category. Number of observations: CNN (115,186), Atlantic (20,136), Telegraph (102,795). The talkativeness null model leads to the emergence of individuals with high IP.
(PNG)

S10 Fig Effect of the constant c on the event rank. The x axis represents the event size. The y axis represents the event rank for an individual with: the highest number of votes (left panel), 10th highest number of votes (right panel). The lines show the relationship between the event size and the event rank for different values of *c*.
(PDF)

S11 Fig Relationship between the IP and the number of events attended. The *x* axis represents the number of events (log scale). The *y* axis represents the IP. Data is pooled from all categories.
(JPG)

S12 Fig Proportion of influencers by number of events. The *x* axis represents the number of events (log scale). Data was binned into intervals of length 10. The *y* axis represents the proportion of individuals with *IP* ≥ 0.8. Data is pooled from all categories.
(PDF)

S13 Fig IP scalling with sample size for a fixed number of ten events. The *x* axis represents the IP decile. The *y* axis represents the mean rejection rate at a cutoff value of 5%. The shape is given by the dataset.
(PDF)

S14 Fig Distribution of votes and event ranks for two individuals with the same mean number of votes. We selected two individuals from the Politics category in the CNN dataset that have a mean of 64 votes per thread and an IP of 0.45 (*left panels*) and 0.94 (*right panels*). The *y* axis represents the frequency. *Upper Panels*: The *x* axis represents the number of votes obtaied per thread. *Lower Panels*: The *x* axis represents the event rank.
(PDF)

S15 Fig Correlation of the IP scores across topic categories. We considered the top 100 individuals with the highest IP in the five largest topic categories. The lower triangle panels show the pairwise scatterplots between the IP scores. The upper triangle panels show the corresponding Pearson correlation coefficient. The diagonal panels show the number of individuals who participated in at least ten events in both categories.
(PDF)

S1 Appendix Talkativeness null model. (PDF)

S2 Appendix Preferential attachement null model. (PDF)

S3 AppendixProof: I(i)∈[0,1],∀i.(PDF)
