# Supplementary figures and images for: Identification of influencers through the wisdom of crowds
Source: PLoS One. 2018 Jul 16;13(7):e0200109. doi: 10.1371/journal.pone.0200109 (PMC6047770; doi:10.1371/journal.pone.0200109)

CNN

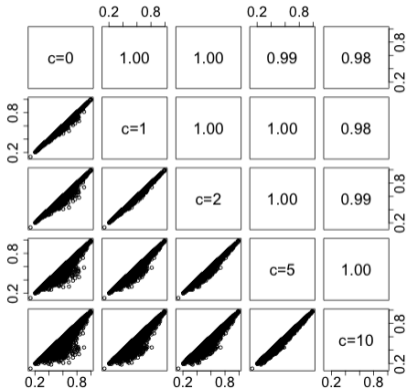

Atlantic

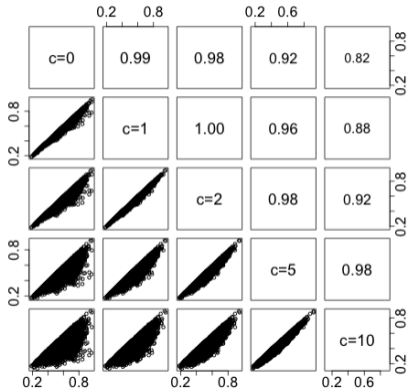

CNN

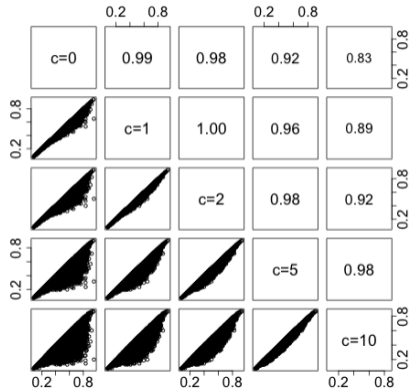

Supplement: S1 Fig — The diagonal panels show the choice of c used to computed the IP. The lower triangle panels show the pairwise scatterplots between the IP scores computed with different c. The upper triangle panels show the corresponding Pearson correlation coefficient. Data is pooled from all topic categories. An individual can be described by multiple data points, each representing his IP in a category where he participated in at least 10 events. Number of observations: CNN (115,186), Atlantic (20,136), Telegraph (102,795). For all three datasets there is a very high pairwise correlation (Pearson r ≥ 0.82) between the IP values for different choices of c. (PDF) [file pone.0200109.s005.pdf]

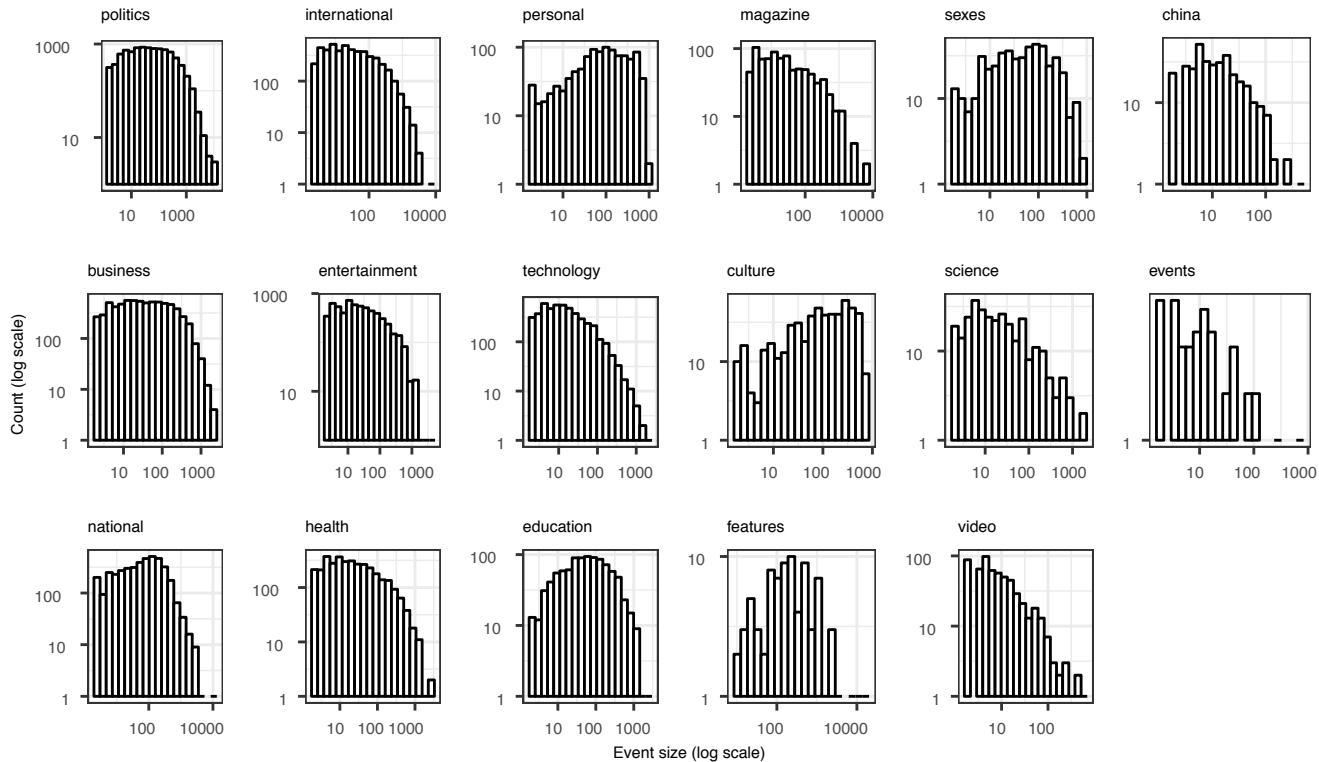

Supplement: S3 Fig — (PDF) [file pone.0200109.s007.pdf]

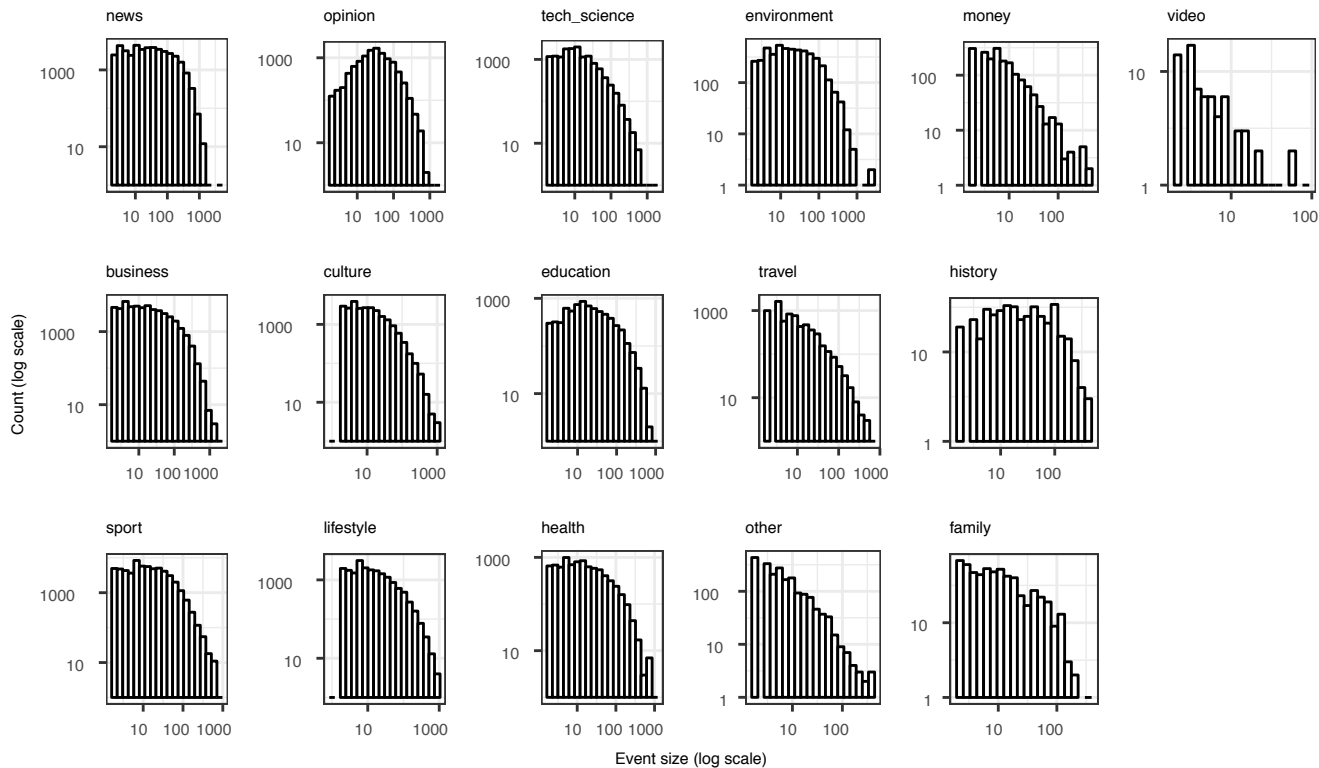

Supplement: S4 Fig — (PDF) [file pone.0200109.s008.pdf]

CNN

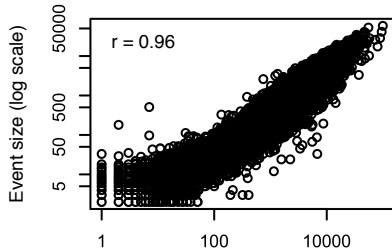

Atlantic

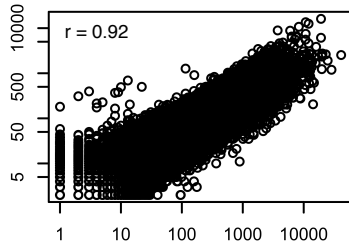

Telegraph

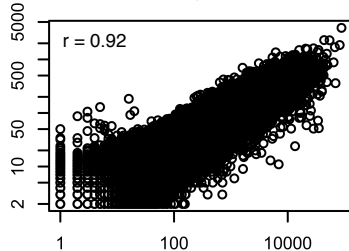

Nr upvotes (log scale)

Supplement: S5 Fig — The x axis represent the total number of votes in the event. The y axis represents the event size. Data is pooled from all categories. Number of observations: CNN (31,035), Atlantic (46,639), Telegraph (268,895). The Spearman correlation coefficient r is computed for the log values. There is a high correlation between the event size and the total number of votes in the event. (PDF) [file pone.0200109.s009.pdf]

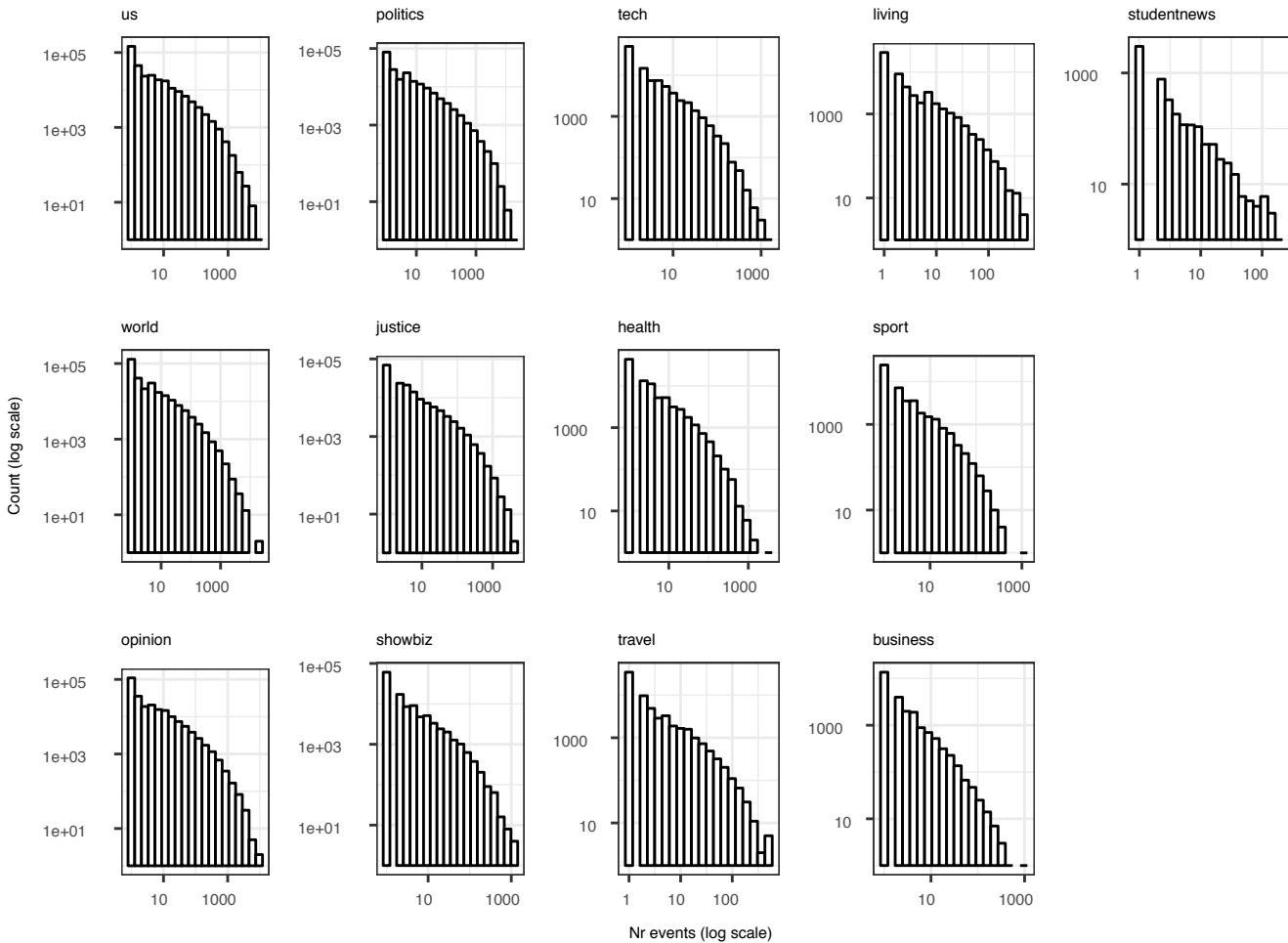

Supplement: S6 Fig — (PDF) [file pone.0200109.s010.pdf]

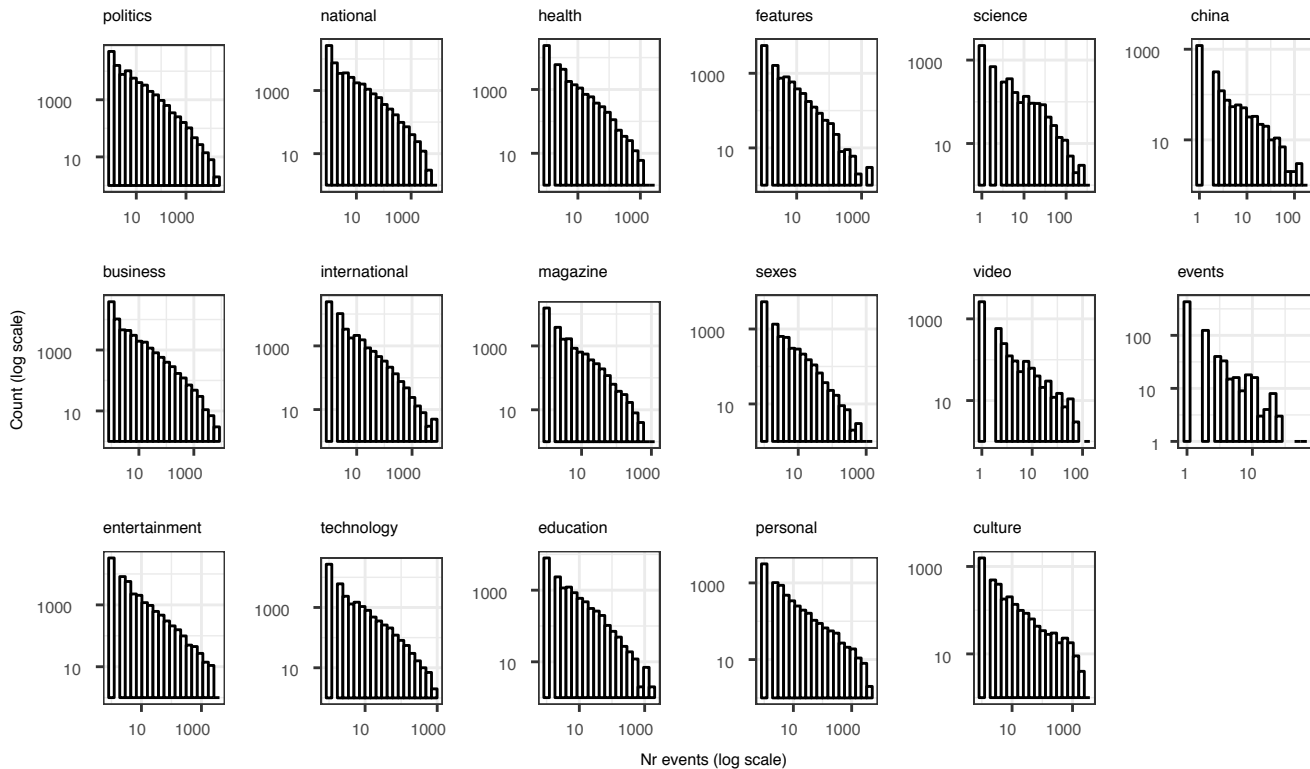

Supplement: S7 Fig — (PDF) [file pone.0200109.s011.pdf]

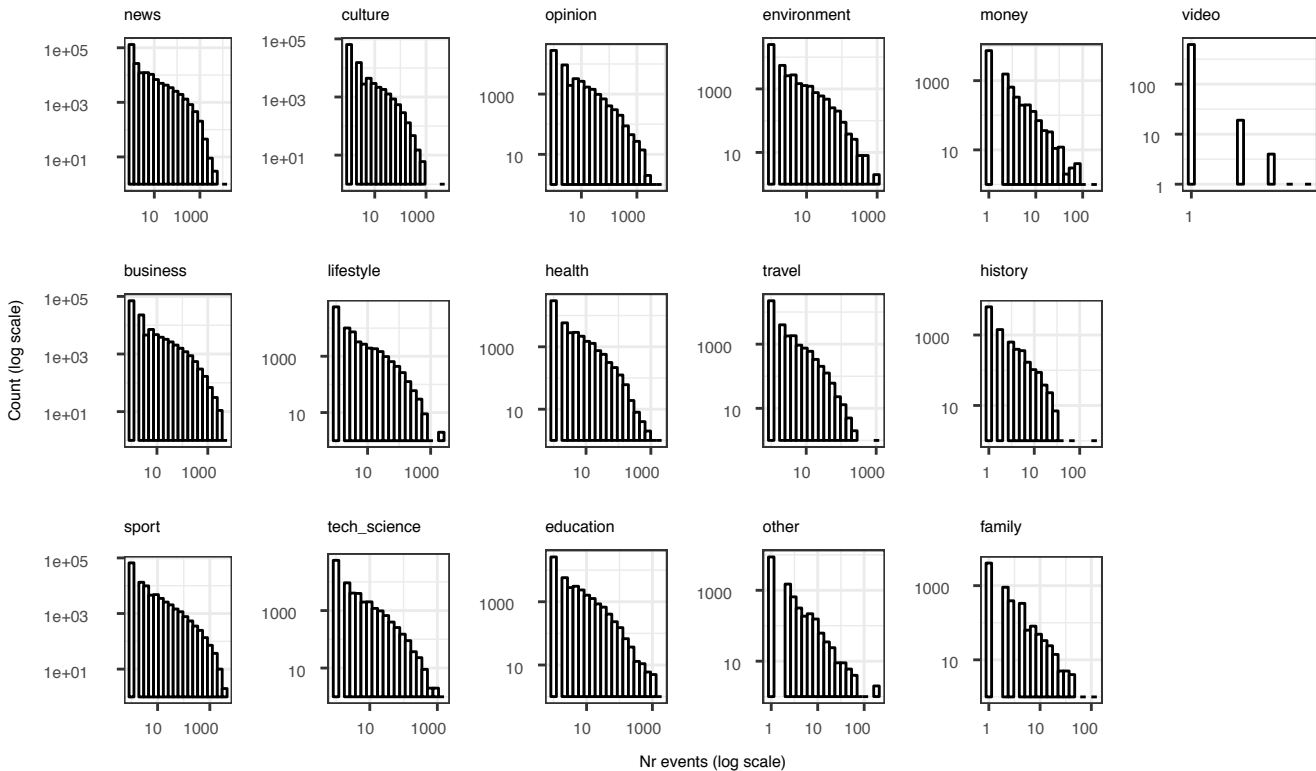

Supplement: S8 Fig — (PDF) [file pone.0200109.s012.pdf]

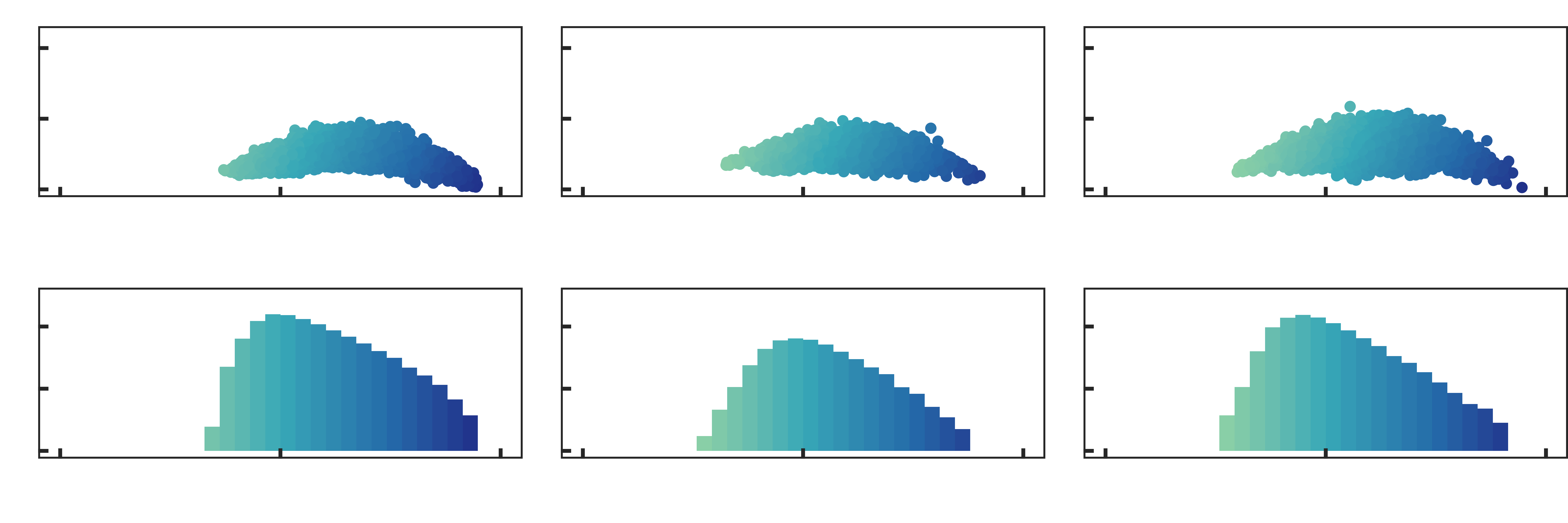

Supplement: S9 Fig — Data is pooled from all categories. An individual can appear in more than one category. Number of observations: CNN (115,186), Atlantic (20,136), Telegraph (102,795). The talkativeness null model leads to the emergence of individuals with high IP. (PNG) [file pone.0200109.s013.png]

Rank=1

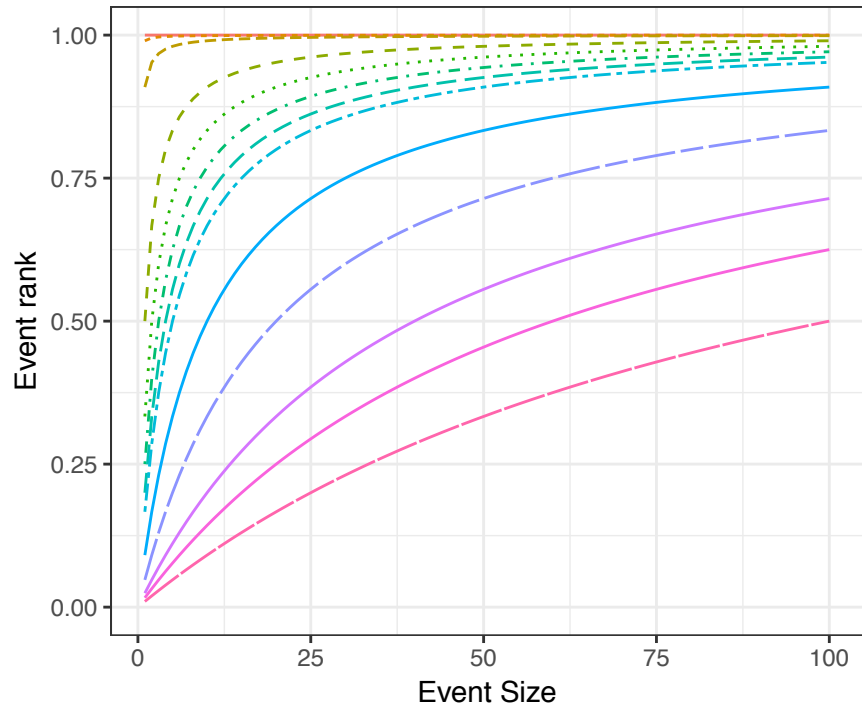Constant  $c$ 

0  
0.01  
0.1  
1  
2  
3  
4  
5  
10  
20  
40  
60  
100

Rank=10

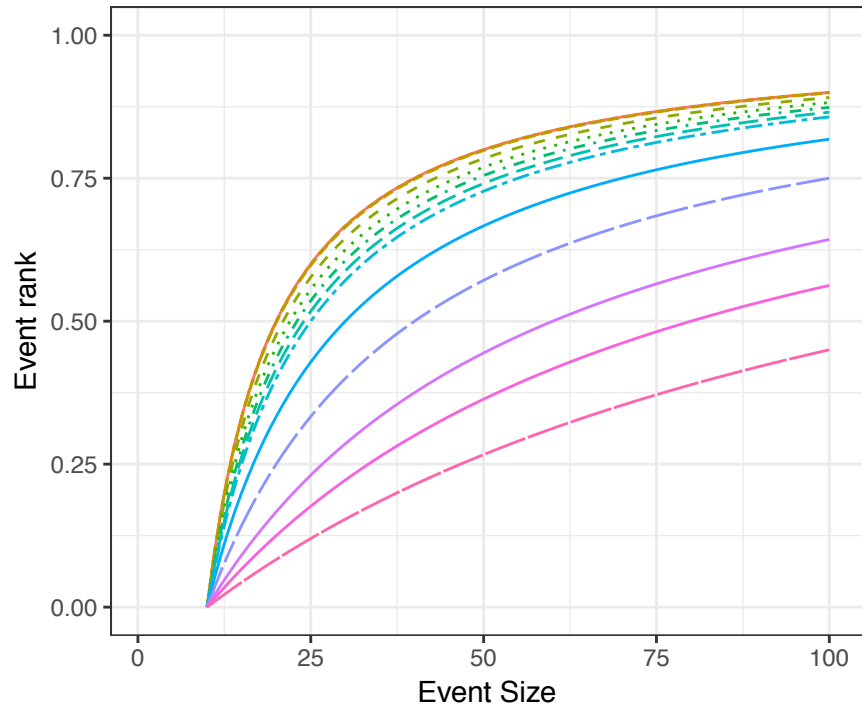Constant  $c$ 

0  
0.01  
0.1  
1  
2  
3  
4  
5  
10  
20  
40  
60  
100

Supplement: S10 Fig — The x axis represents the event size. The y axis represents the event rank for an individual with: the highest number of votes (left panel), 10th highest number of votes (right panel). The lines show the relationship between the event size and the event rank for different values of c. (PDF) [file pone.0200109.s014.pdf]

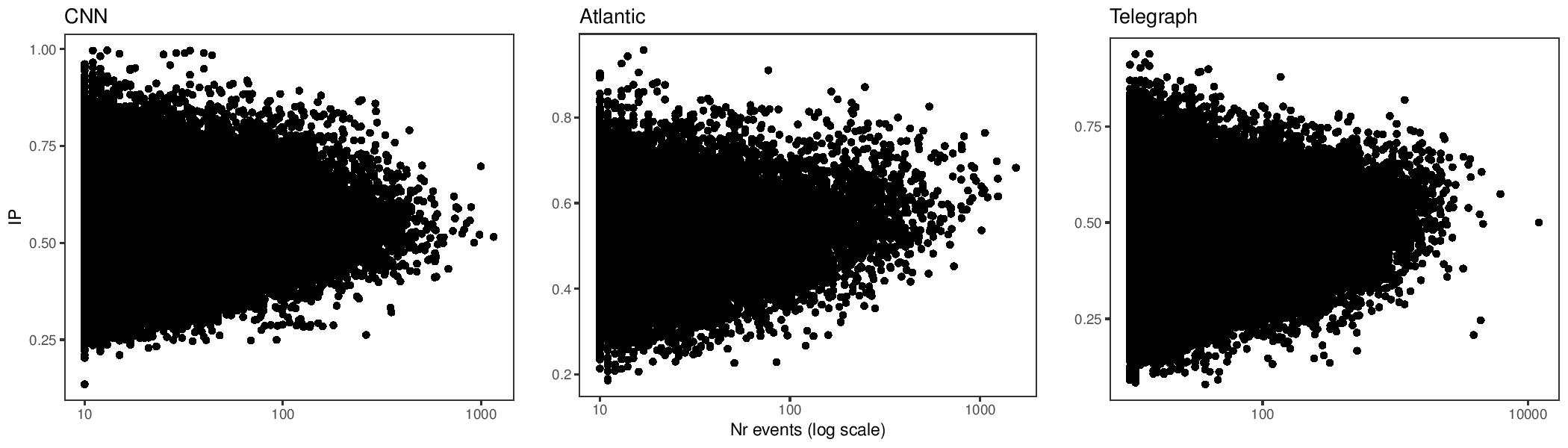

Supplement: S11 Fig — The x axis represents the number of events (log scale). The y axis represents the IP. Data is pooled from all categories. (JPG) [file pone.0200109.s015.jpg]

**CNN**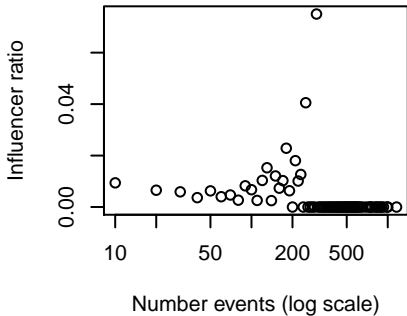**Atlantic**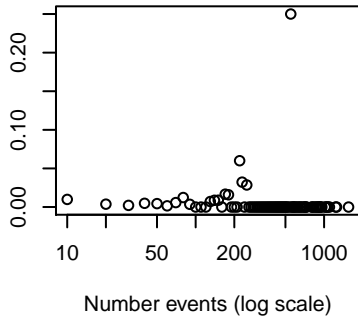

Supplement: S12 Fig — The x axis represents the number of events (log scale). Data was binned into intervals of length 10. The y axis represents the proportion of individuals with IP ≥ 0.8. Data is pooled from all categories. (PDF) [file pone.0200109.s016.pdf]

Mean rejection rate

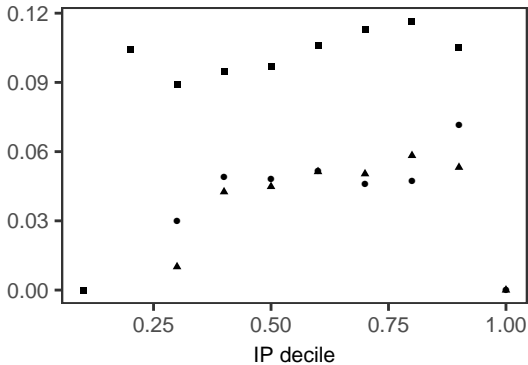

Dataset

• Atlantic

▲ CNN

■ Telegraph

Supplement: S13 Fig — The x axis represents the IP decile. The y axis represents the mean rejection rate at a cutoff value of 5%. The shape is given by the dataset. (PDF) [file pone.0200109.s017.pdf]

**Low IP**

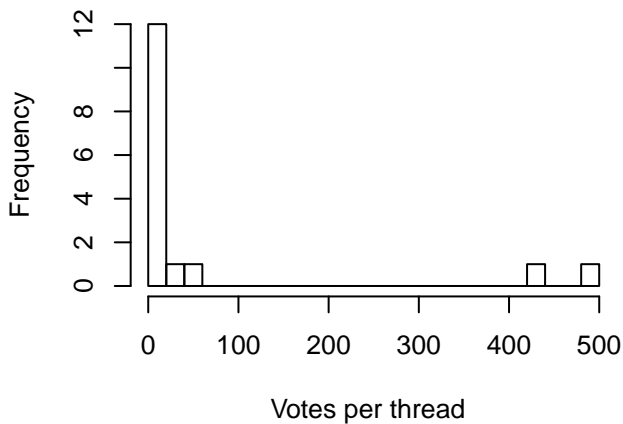

**High IP**

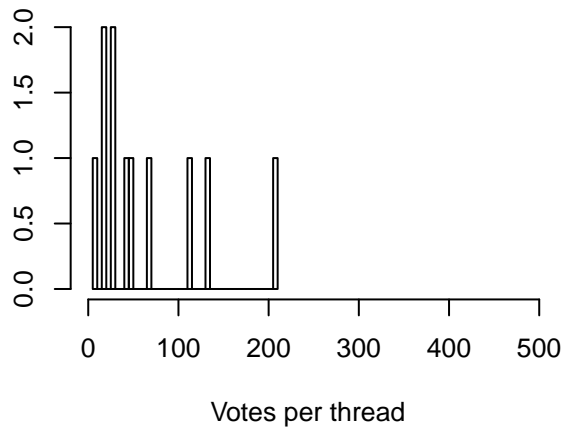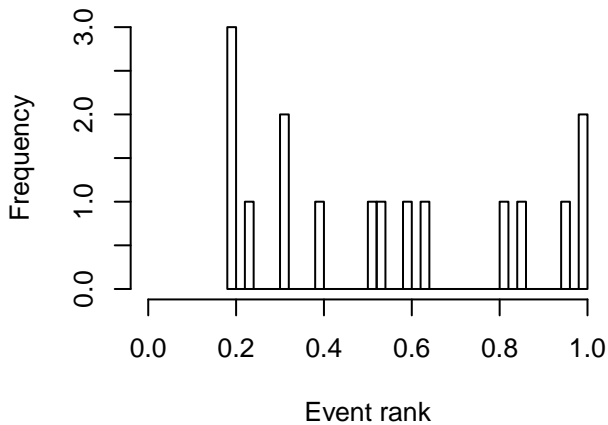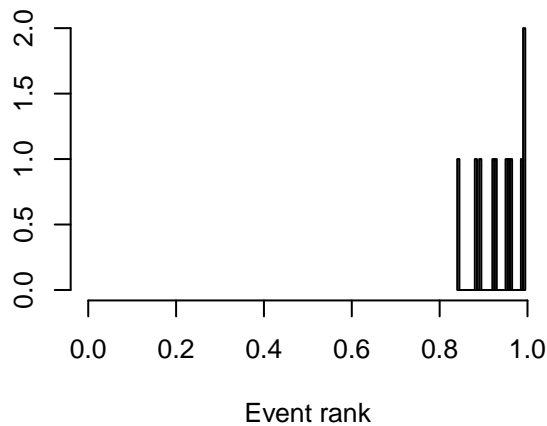

Supplement: S14 Fig — We selected two individuals from the Politics category in the CNN dataset that have a mean of 64 votes per thread and an IP of 0.45 (left panels) and 0.94 (right panels). The y axis represents the frequency. Upper Panels: The x axis represents the number of votes obtaied per thread. Lower Panels: The x axis represents the event rank. (PDF) [file pone.0200109.s018.pdf]

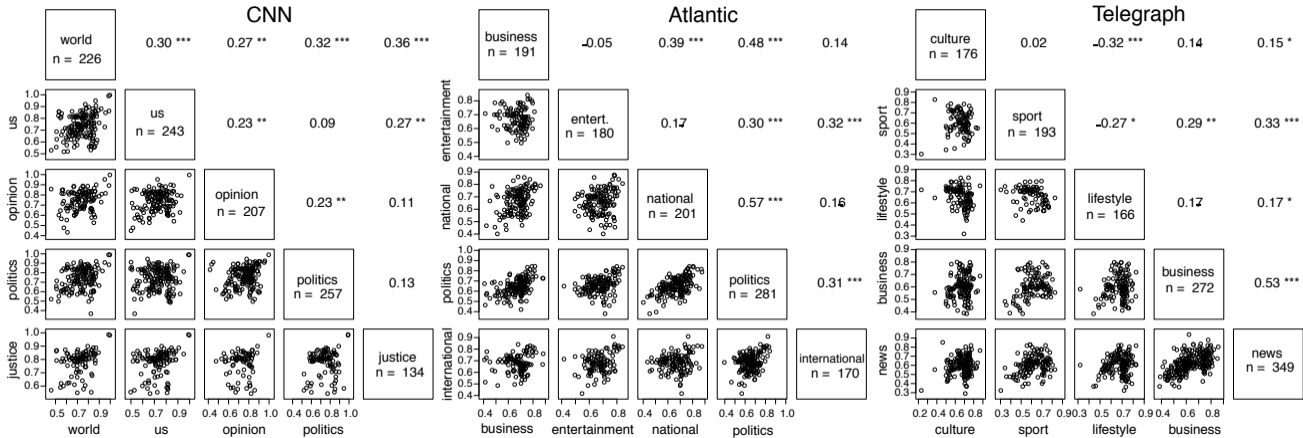

Supplement: S15 Fig — We considered the top 100 individuals with the highest IP in the five largest topic categories. The lower triangle panels show the pairwise scatterplots between the IP scores. The upper triangle panels show the corresponding Pearson correlation coefficient. The diagonal panels show the number of individuals who participated in at least ten events in both categories. (PDF) [file pone.0200109.s019.pdf]
