# Supplementary material for: Identification of influencers through the wisdom of crowds
Source: PLoS One. 2018 Jul 16;13(7):e0200109. doi: 10.1371/journal.pone.0200109 (PMC6047770; doi:10.1371/journal.pone.0200109)
Supplement: S2 Fig — (PDF) [file pone.0200109.s006.pdf]

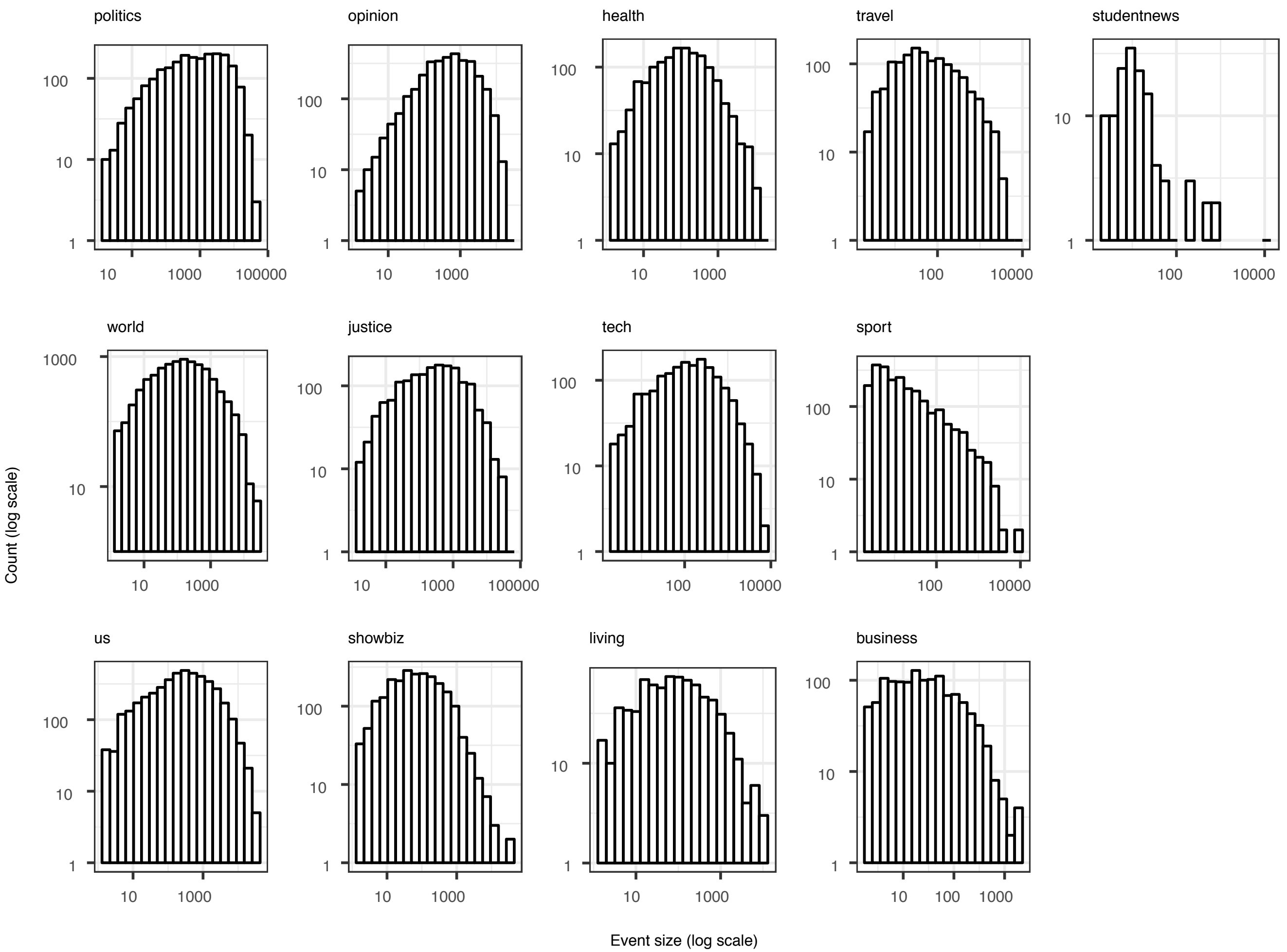
politics
opinion
health
travel
studentnews
world
justice
tech
sport
us
showbiz
living
business
Count (log scale)
Event size (log scale)
