# Supplementary material for: Identification of influencers through the wisdom of crowds
Source: PLoS One. 2018 Jul 16;13(7):e0200109. doi: 10.1371/journal.pone.0200109 (PMC6047770; doi:10.1371/journal.pone.0200109)
Supplement: S1 Appendix — (PDF) [file pone.0200109.s020.pdf]

Talkativeness null model

To test if data can be explained by the talkativeness effect, we create a null model in which all posts in a thread are equally likely to receive a vote. Thus the probablity of a post j to receive a vote is:

$$p(j) = \frac{1}{K}$$

where K is the total number of posts in the thread. To compute the number of votes received by each post under the null model, we sample with replacement from all posts a number of times equal to the observed number of votes in the thread. Then we compute the IP using the sum of randomized vote scores as input. The procedure is repeated 100 times and the IP under the null model is computed as the mean IP over the repetitions.
