# Supplementary material for: Identification of influencers through the wisdom of crowds
Source: PLoS One. 2018 Jul 16;13(7):e0200109. doi: 10.1371/journal.pone.0200109 (PMC6047770; doi:10.1371/journal.pone.0200109)
Supplement: S2 Appendix — (PDF) [file pone.0200109.s021.pdf]

Preferential attachement null model

To test if data can be explained by the rich-get-richer effect we create a null model in which the probability of a post to receive a vote depends on how many votes the post had so far received. We consider the observed number of votes in a thread was distributed across the posts in the thread at $\tau = 10$ points in time. The time points are select uniformly at random from the time-span of the thread. The number of votes being distributed is the same at all time points (the total number of votes in the thread divided by τ). We compute the probability of a post j written until time s to receive a vote distributed at s as:

$$p(j,s) = \alpha \frac{1}{\sum\limits_{k=1}^{s} q(k)} + (1-\alpha) \frac{v(j,s)}{\sum\limits_{k=1}^{s} v(k)}$$

where $\sum_{k=1}^{s} q(k)$ is the number of posts written until s, $v(j,s)$ the number of votes of post j at time s and $\sum_{k=1}^{s} v(k)$ the total number of votes observed until time s. Thus, at time s, with probability α a vote is given to a post sampled uniformly from all posts written until s and with probability $1-\alpha$ the vote is given to one of the posts written until s sampled with probability determined by preferential attachement. The IP is computed using the sum of randomized vote scores as input. The procedure is repeated 100 times and the IP under the null model is computed as the mean IP over the repetitions.
