# Supplementary material for: Identification of influencers through the wisdom of crowds
Source: PLoS One. 2018 Jul 16;13(7):e0200109. doi: 10.1371/journal.pone.0200109 (PMC6047770; doi:10.1371/journal.pone.0200109)
Supplement: S3 Appendix — (PDF) [file pone.0200109.s022.pdf]

Proof: $\mathcal{I}(i) \in [0,1], \forall i$

Proposition 1 *For any vector with elements in the unit interval, the mean is always greater than or equal to the variance.*

Proof 1 *Let* $\mathbf{X} = \{x_1, \ldots, x_n\}$ *be a vector with:* $x_k \in [0,1], \forall k = 1, \ldots, n$.
If the mean ($\langle \mathbf{X} \rangle$) *is greater than or equal to the variance* ($\langle \mathbf{X}^2 \rangle - \langle \mathbf{X} \rangle^2$) *it follows that:*

$$\langle \mathbf{X} \rangle + \langle \mathbf{X} \rangle^2 \geq \langle \mathbf{X}^2 \rangle$$

The inequality always holds as all x *values are in the unit interval* ($x_k \in [0,1]$) *and thus the square is always greater than or equal to the original value* ($x_k \geq x_k^2, \forall k = 1, \ldots, n$).
